# Potential of Pandan Root and Teak Leaf Extracts in Managing Maternal Hyperglycemia During Pregnancy: Comparative Efficacy and Mechanistic Insights

**DOI:** 10.3390/ijms26125506

**Published:** 2025-06-09

**Authors:** Sasitorn Kerdsuknirund, Panida Khunkaewla, Pakanit Kupittayanant, Suthida Chanlun, Pattama Tongdee, Porntip Nimkuntod, Sajeera Kupittayanant

**Affiliations:** 1 School of Preclinical Sciences, Institute of Science, Suranaree University of Technology, Nakhon Ratchasima 30000, Thailand; sasi.kerdsuknirund@gmail.com; 2 Biochemistry-Electrochemistry Research Unit, School of Chemistry, Institute of Science, Suranaree University of Technology, Nakhon Ratchasima 30000, Thailand; kpanida@sut.ac.th; 3 School of Animal Technology and Innovation, Institute of Agricultural Technology, Suranaree University of Technology, Nakhon Ratchasima 30000, Thailand; pakanit@sut.ac.th; 4 Department of Pathobiology, Faculty of Veterinary Medicine, Khon Kaen University, Khon Kaen 40000, Thailand; sutvir@kku.ac.th; 5 School of Obstetrics and Gynecology, Institute of Medicine, Suranaree University of Technology, Nakhon Ratchasima 30000, Thailand; pattama_t@sut.ac.th; 6 Suranaree University of Technology Hospital, Nakhon Ratchasima 30000, Thailand; porntipnimk@sut.ac.th; 7 School of Internal Medicine, Institute of Medicine, Suranaree University of Technology, Nakhon Ratchasima 30000, Thailand

**Keywords:** gestational diabetes mellitus, teak leaf extract, pandan root extract, streptozotocin-induced diabetes, maternal hyperglycemia

## Abstract

Maternal hyperglycemia during pregnancy poses significant health risks to both mother and fetus. Although gestational diabetes mellitus (GDM) is mainly characterized by insulin resistance, severe hyperglycemia may also result from impaired pancreatic function. This study evaluates the therapeutic potential of pandan (*Pandanus amaryllifolius*) root and teak (*Tectona grandis*) leaf extracts in managing streptozotocin (STZ)-induced maternal hyperglycemia in pregnant rats, compared to metformin. Methods: Pregnant rats were administered STZ (60 mg/kg) on gestation day 5. Treatments with metformin (300 mg/kg), pandan extract (low, medium, high doses), and teak extract (low, medium, high doses) were given from gestation day 7 to 21. The key parameters included the maternal blood glucose, insulin levels, pancreatic morphology, fetal and placental outcomes, and gas chromatography/mass spectrometry (GC/MS) phytochemical profiling. GC/MS analysis identified 2,3-butanediol and propanoic acid derivatives as major compounds in pandan, while teak contained catavic acid and methyl copalate. The high-dose pandan extract significantly reduced the maternal blood glucose (*p* < 0.05), improved the insulin levels and pancreatic mass index, and increased the number of live fetuses, with effects comparable to metformin. The teak extract showed milder improvements. The pandan extract demonstrated dose-dependent antidiabetic potential in this STZ-induced model. Future studies should evaluate these effects in insulin-resistance-based GDM models.

## 1. Introduction

Gestational diabetes mellitus (GDM) is a metabolic disorder characterized by impaired glucose regulation during pregnancy, primarily due to progressive insulin resistance (IR) and inadequate compensatory insulin secretion. Unlike type 1 diabetes, which results from autoimmune beta-cell destruction, GDM arises when pregnancy-related hormonal changes lead to reduced insulin sensitivity [1]. This condition increases the risk of maternal complications, such as preeclampsia and cesarean delivery, as well as fetal complications, including macrosomia and neonatal hypoglycemia. Additionally, mothers diagnosed with GDM have a higher likelihood of developing type 2 diabetes later in life [2,3].

The management of GDM typically involves dietary modifications, physical activity, and pharmacological interventions such as insulin and oral hypoglycemic agents [4]. While insulin therapy is effective, it requires frequent monitoring and carries a risk of maternal hypoglycemia [5]. Oral hypoglycemic agents, such as metformin, have shown promise in managing GDM but require careful assessment due to the potential effects on fetal development [6]. These limitations have led to increasing interest in alternative and complementary therapies, including medicinal plants with hypoglycemic properties.

Medicinal plants have been widely studied for their glucose-lowering effects and potential role in improving insulin sensitivity [7]. Various natural remedies, including fenugreek, bitter melon, cinnamon, and aloe vera, have been explored for their use in diabetes management [8]. Among these, pandan (*Pandanus amaryllifolius* Roxb.) and teak (*Tectona grandis* L.f.) are notable for their traditional use in Southeast Asian medicine for managing hyperglycemia [9,10].

Pandan has been reported to exhibit antihyperglycemic, antioxidant, and anti-inflammatory properties, attributed to its bioactive compounds, including alkaloids, terpenoids, flavonoids, saponins, anthraquinone glycosides, and cardiac glycosides [11]. Studies have shown that pandan leaf extract inhibits α-amylase activity, enhances glucose uptake, and scavenges free radicals, all of which contribute to improving glycemic control and insulin sensitivity [12]. Additionally, pandan root extract has been found to increase hepatic glycogen storage and insulin secretion, leading to lower blood glucose levels in diabetic models [13]. The incorporation of pandan leaf extract into parboiled rice has been shown to lower its glycemic index and reduce the postprandial blood glucose levels in healthy individuals, supporting its potential use as a dietary intervention for diabetes [14]. In traditional Thai medicine, pandan roots are used as a natural remedy for diabetes, with the decoction of boiled pandan roots commonly consumed to reduce blood glucose levels [15]. While pandan has been widely used for general health benefits, its specific applications in pregnancy-related hyperglycemia remain underexplored [16].

Teak has also been traditionally used to regulate blood glucose levels and metabolic disorders [17]. Teak leaves contain naphthoquinones, anthraquinones, terpenoids, flavonoids, steroids, saponins, and phenolic compounds, many of which exhibit antioxidant, anti-inflammatory, and hypoglycemic effects [17]. A preclinical study demonstrated that teak leaf extract can significantly lower the blood glucose levels in streptozotocin (STZ)-induced diabetic rats, indicating its potential antidiabetic properties [10]. Teak leaves are traditionally prepared as a decoction and consumed to regulate blood glucose levels [18]. In Thai traditional medicine, pandan and teak are sometimes used in combination for diabetes management, suggesting possible synergistic effects [18]. Moreover, a clinical trial found that consuming 225 mL of pandan leaf decoction daily for seven days significantly reduced the blood glucose levels in diabetic patients, demonstrating potential efficacy in human populations [19]. However, the specific effects of pandan and teak extracts on pregnancy-related hyperglycemia remain unclear.

Although previous studies have reported the antidiabetic properties of pandan and teak extracts, their role in managing hyperglycemia during pregnancy remains unexplored. Most existing research focuses on type 2 diabetes and general hyperglycemia, with limited evidence of their effects in terms of pregnancy-induced insulin resistance and maternal glucose metabolism [20]. While GDM is primarily characterized by insulin resistance [20], our study investigates STZ-induced maternal hyperglycemia, which primarily results from beta-cell dysfunction rather than insulin resistance. We acknowledge that this model does not fully replicate the pathophysiology of human GDM; however, it allows for the evaluation of hyperglycemia-related pregnancy complications and the potential effects of plant-based interventions on maternal and fetal health.

This study aims to evaluate the therapeutic potential of pandan root and teak leaf extracts in maternal hyperglycemia, pancreatic function, and fetal outcomes. By comparing these extracts with metformin, a well-established treatment for gestational diabetes, we aim to determine whether these natural interventions offer comparable benefits in terms of glycemic control and fetal health. Additionally, our study investigates whether these extracts can modulate insulin levels, improve placental function, and enhance fetal viability in a hyperglycemic pregnancy model.

## 2. Results

### 2.1. Phytochemical Composition of Pandan and Teak Extracts

**Figure 1:**
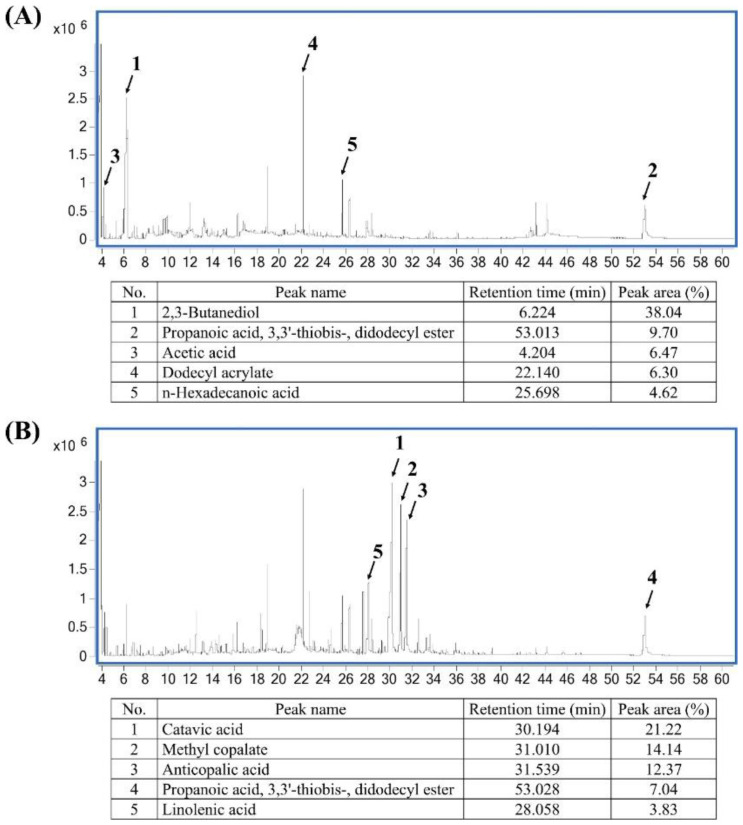
Gas chromatography–mass spectrometry (GC/MS) chromatograms and corresponding identified compounds. (**A**) GC/MS chromatogram and compound identification for pandan (*Pandanus amaryllifolius*) root extract. Peaks correspond to identified compounds, with their retention times and peak areas listed in the accompanying table. The major identified compounds include 2,3-butanediol (retention time: 6.224 min, peak area: 38.04%) and propanoic acid, 3,3′-thiobis-, dodecyl ester (retention time: 53.013 min, peak area: 9.70%). (**B**) GC/MS chromatogram and compound identification for teak (*Tectona grandis*) leaf extract. Identified compounds include catavic acid (retention time: 30.194 min, peak area: 21.22%) and methyl copalate (retention time: 31.010 min, peak area: 14.14%). Peaks are numbered in correspondence with the table below each chromatogram. Labeled peaks indicate the identified compounds in each sample. The *x*-axis represents the retention time (minutes), and the *y*-axis represents the signal intensity.

The GC/MS chromatograms of the pandan and teak extracts are shown in Figure 1A and Figure 1B, respectively. The top five most abundant compounds in the pandan extract were 2,3-butanediol (38.04%), propanoic acid, 3,3′-thiobis-, didodecyl ester (9.70%), acetic acid (6.47%), dodecyl acrylate (6.30%), and n-hexadecanoic acid (4.62%). In the teak extract, the predominant compounds included catavic acid (21.22%), methyl copalate (14.14%), anticopalic acid (12.37%), propanoic acid, 3,3′-thiobis-, didodecyl ester (7.04%), and linolenic acid (3.83%). These bioactive compounds suggest potential antidiabetic and antioxidant properties. A list of all compounds identified in the GC/MS analysis is provided in the Supplementary Materials.

### 2.2. Induction of Maternal Hyperglycemia

**Figure 2:**
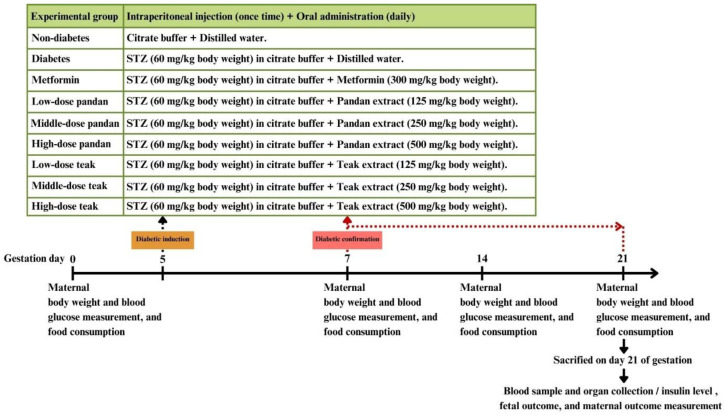
Experimental design and treatment schedule for gestational hyperglycemia induction and intervention in pregnant rats. Pregnant rats were divided into experimental groups based on the treatments, including a non-diabetic control, diabetic control, metformin-treated, and various doses of pandan and teak extracts. Gestational hyperglycemia was induced using intraperitoneal injection of streptozotocin (STZ) at 60 mg/kg in citrate buffer on gestation day 5. Diabetic confirmation was conducted on gestation day 7, after which daily oral administration of the treatments (metformin, pandan extract, or teak extract) was initiated. The maternal body weight (BW), blood glucose levels, and food consumption were monitored at gestation days 0, 7, 14, and 21. On gestation day 21, the animals were sacrificed for the pregnancy outcome assessment, blood sample collection, and insulin level measurement.

The experimental designs are illustrated in Figure 2.

**Figure 3:**
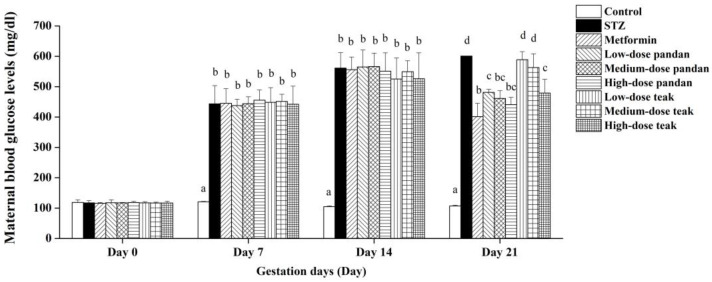
Maternal blood glucose levels (mg/dL) during pregnancy in different experimental groups. The blood glucose levels were measured on gestation days 0, 7, 14, and 21 in pregnant rats assigned to different treatment groups: control (non-diabetic), streptozotocin (STZ)-induced hyperglycemic (untreated), metformin-treated, and varying doses of pandan and teak extracts. On gestation day 0, all the groups showed similar baseline blood glucose levels. By gestation day 7, STZ administration significantly increased the blood glucose levels in all the experimental groups compared to the control (*p* < 0.05). The blood glucose remained elevated in untreated hyperglycemic rats (STZ group), while metformin and high doses of pandan and teak extracts exhibited a trend of glucose reduction by gestation days 14 and 21. Groups labeled with different superscript letters denote statistically significant differences (*p* < 0.05; *n* = 5 per group). Data are expressed as the mean ± standard deviation (SD) and were analyzed using one-way ANOVA followed by Tukey’s post hoc test (*p* < 0.05).

Figure 3 confirms the successful induction of maternal hyperglycemia using STZ, as the blood glucose levels in the STZ group increased significantly from gestation day 7 (443.80 ± 59.73 mg/dL) to day 14 (561.40 ± 50.53 mg/dL) and day 21 (601.00 ± 0.00 mg/dL) compared to the control group (*p* < 0.05).

In the metformin-treated and extract-treated groups, the blood glucose levels also increased significantly from gestation day 7 (400–500 mg/dL) to day 14 (400–600 mg/dL) compared to the control group (*p* < 0.05). However, there was no statistical difference between the extract groups and the STZ group (*p* > 0.05) during this period.

By gestation day 21, metformin and all the doses of pandan extract significantly reduced the blood glucose levels (400–500 mg/dL) compared to the STZ group (>500 mg/dL, *p* < 0.05). Although the levels remained significantly higher than those in the control group (<100 mg/dL, *p* < 0.05), this reduction suggests an antidiabetic effect. Similarly, the high-dose teak extract group (400–500 mg/dL) exhibited a decreasing trend (*p* > 0.05), while the low- and medium-dose teak extract groups (500–600 mg/dL) did not significantly differ from the STZ group (*p* > 0.05).

### 2.3. Maternal Body Weight

**Figure 4:**
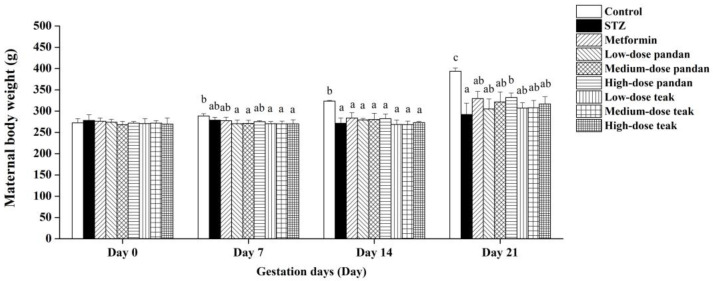
Maternal body weight (g) during pregnancy in the different experimental groups. The maternal body weight was measured on gestation days 0, 7, 14, and 21 in pregnant rats assigned to the different treatment groups: control (non-diabetic), streptozotocin (STZ)-induced hyperglycemic (untreated), metformin-treated, and varying doses of pandan and teak extracts. On gestation days 0 and 7, the maternal body weights across all the groups remained similar and within the baseline range. By gestation day 14, STZ administration significantly decreased the maternal body weight in all the experimental groups compared to the control group (*p* < 0.05). High-dose pandan extract significantly increased the maternal body weight on gestation day 21 compared to the untreated hyperglycemic rats (STZ group, *p* < 0.05). Groups labeled with different superscript letters denote statistically significant differences at the same time point (*p* < 0.05; *n* = 5 per group). Data are expressed as the mean ± standard deviation (SD) and were analyzed using one-way ANOVA followed by Tukey’s post hoc test (*p* < 0.05).

The maternal body weight gradually decreased in the STZ group compared to the control group, with a significant reduction observed on gestation day 14 (*p* < 0.05; Figure 4). While the metformin-treated and extract-treated groups showed an increasing trend, the differences were not statistically significant when compared to the STZ group (*p* > 0.05). Interestingly, the rats receiving high-dose pandan extract exhibited a significant increase in maternal body weight on gestation day 21 compared to the untreated hyperglycemic rats (*p* < 0.05).

### 2.4. Maternal Insulin Levels

**Figure 5:**
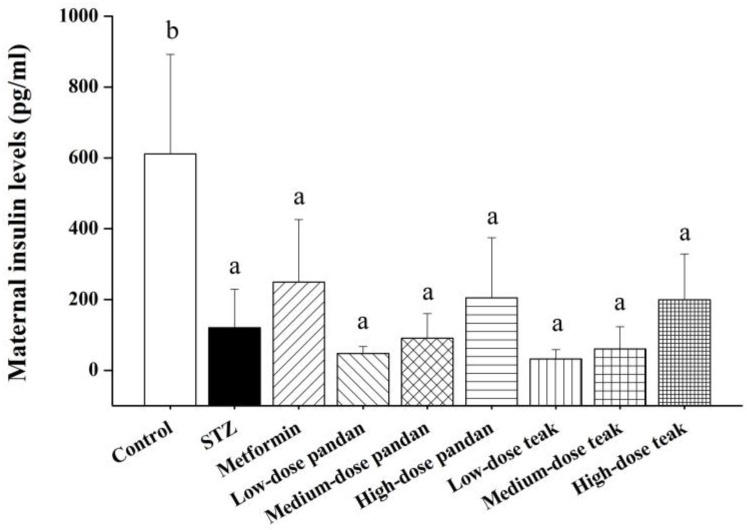
Maternal insulin levels (pg/mL) at gestation day 21 in the different experimental groups. The insulin levels were measured in pregnant rats across the treatment groups: control (non-diabetic), streptozotocin (STZ)-induced hyperglycemic (untreated), metformin-treated, and varying doses of pandan and teak extracts. The control group exhibited significantly higher insulin levels compared to all the other groups (*p* < 0.05), while the STZ-induced hyperglycemic rats and treated groups showed lower insulin levels, with no significant differences among the treatments. Groups labeled with different superscript letters denote statistically significant differences (*p* < 0.05; *n* = 5 per group). Data are expressed as the mean ± standard deviation (SD) and were analyzed using one-way ANOVA followed by Tukey’s post hoc test (*p* < 0.05).

The maternal insulin levels (Figure 5) were significantly lower in the STZ group compared to the control group (*p* < 0.05). While the insulin levels in the metformin-treated and extract-treated groups were higher than in the STZ group, the increase was not statistically significant (*p* > 0.05). Interestingly, the high-dose pandan and teak extracts exhibited an increasing trend, with the insulin levels approaching those seen in the metformin group.

### 2.5. Effects on Pancreatic Morphology

**Figure 6:**
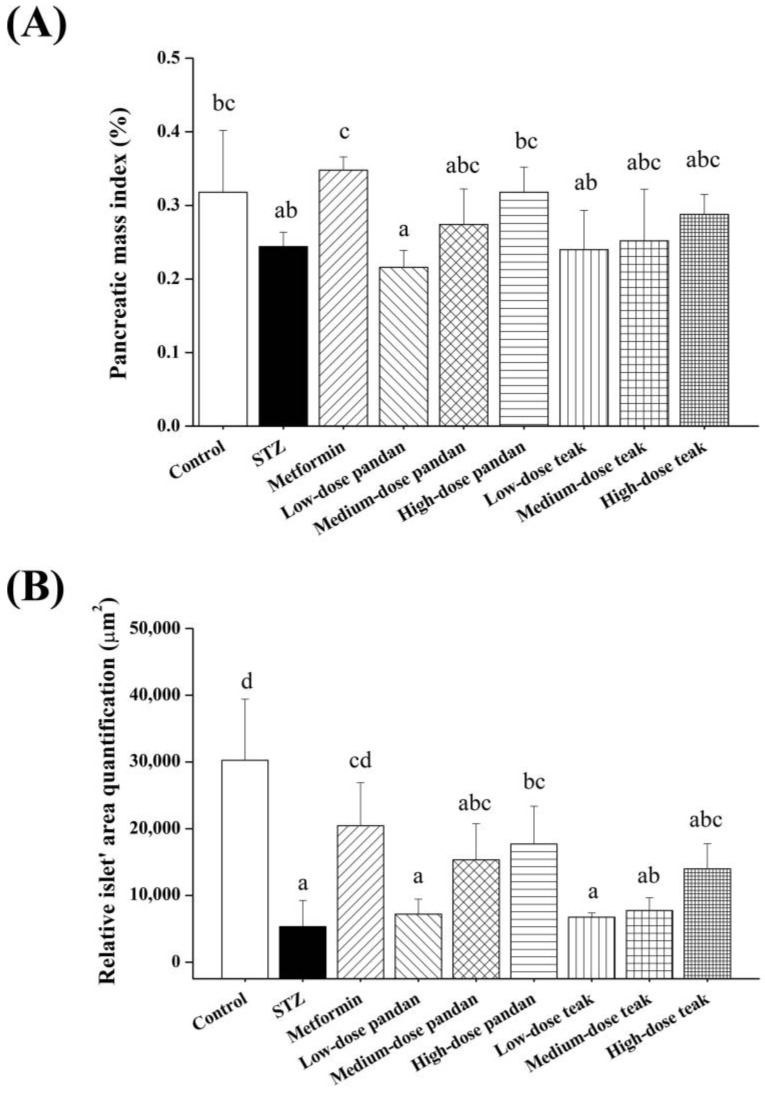
Pancreatic mass index and relative islet area quantification in the different experimental groups at gestation day 21. (**A**) Pancreatic mass index (%). The pancreatic mass index was calculated as the ratio of pancreatic weight to body weight in pregnant rats from the different treatment groups: control (non-diabetic), streptozotocin (STZ)-induced hyperglycemic (untreated), metformin-treated, and varying doses of pandan and teak extracts. The STZ group exhibited the lowest pancreatic mass index, whereas the medium-dose pandan group showed a significant increase compared to STZ (*p* < 0.05). (**B**) Relative islet area quantification (µm^2^). The pancreatic islet area was measured to assess the β-cell preservation among groups. The control group showed the largest islet area, while the STZ-induced hyperglycemic group exhibited a significant reduction. Treatment with metformin, medium-dose pandan, and high-dose teak extracts resulted in partial islet preservation compared to STZ (*p* < 0.05). Groups labeled with different superscript letters denote statistically significant differences (*p* < 0.05; *n* = 5 per group). Data are expressed as the mean ± standard deviation (SD) and were analyzed using one-way ANOVA followed by Tukey’s post hoc test (*p* < 0.05).

Figure 6A illustrates that STZ significantly reduced the pancreatic mass index compared to the control group (*p* < 0.05). As expected, the pancreatic mass index was significantly restored in the metformin group (*p* < 0.05), reaching values similar to the control group (*p* > 0.05). Notably, the medium-dose and high-dose pandan and teak extract groups also showed pancreatic mass index values comparable to metformin (*p* > 0.05).

**Figure 7:**
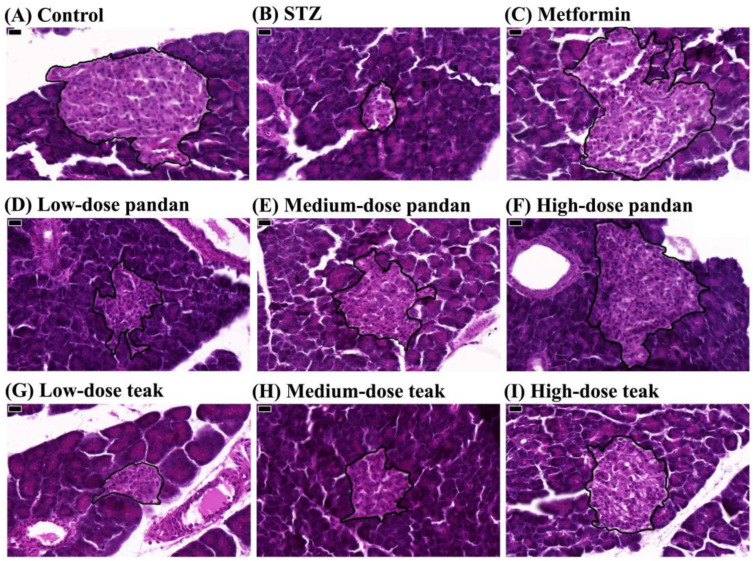
Histological analysis of pancreatic islets in the different experimental groups. Representative hematoxylin and eosin (H&E)-stained photomicrographs of pancreatic islets from pregnant rats at 400× magnification (scale bar: 20 μm). Pancreatic islets are outlined in black. (**A**) Control group: normal islet architecture with well-preserved β-cell distribution. (**B**) Streptozotocin (STZ)-induced hyperglycemic group: marked reduction in islet size and disrupted cellular arrangement. (**C**) Metformin-treated group: partial preservation of islet structure and increased β-cell density. (**D**–**F**) Pandan extract-treated groups: dose-dependent improvement in islet structure, with higher doses showing greater preservation. (**G**–**I**) Teak extract-treated groups: improved islet morphology in higher doses compared to STZ-induced hyperglycemia. The islet size and morphology were used as indicators of gestational hyperglycemia-associated pancreatic changes.

As shown in Figure 6B, the relative islet area was significantly reduced in the STZ group compared to the control group (*p* < 0.05). However, the metformin group and the high-dose pandan extract group exhibited significant increases in the relative islet area compared to the STZ group (*p* < 0.05), suggesting potential preservation of the pancreatic architecture. Histological examination of pancreatic tissue (Figure 7) confirmed that STZ-induced hyperglycemia led to structural deterioration of the pancreatic islets, contributing to the observed decrease in the islet area. Both the pandan and teak extracts demonstrated remodeling effects, with the high-dose treatment groups showing the most pronounced restoration of islet morphology.

### 2.6. Fetal and Placental Outcomes

**Figure 8:**
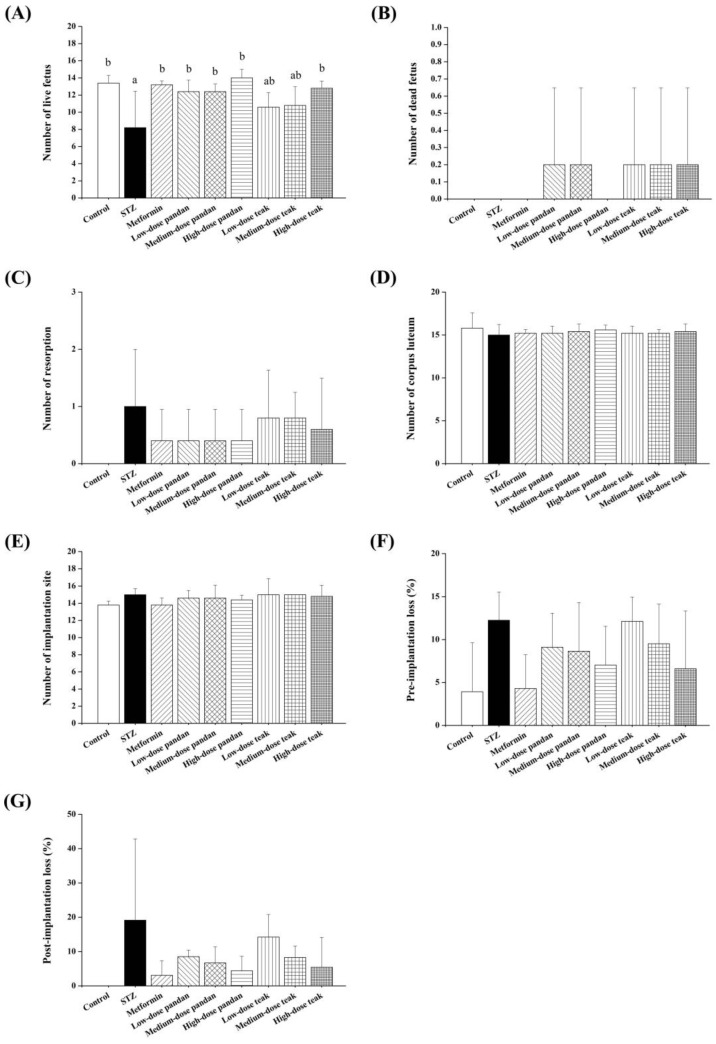
Pregnancy outcomes in different experimental groups at gestation day 21. (**A**) Number of live fetuses: the streptozotocin (STZ)-induced hyperglycemic group exhibited a lower number of live fetuses compared to the control and treatment groups. Treatment with pandan and teak extracts, particularly at medium and high doses, improved fetal survival (*p* < 0.05). (**B**) Number of dead fetuses: the STZ-induced hyperglycemic group showed a higher number of dead fetuses, though the variations among treatment groups were not statistically significant. (**C**) Number of resorptions: resorption rates were elevated in the STZ-induced group, while the pandan and teak extract treatments showed a trend of reduction. (**D**) Number of corpora lutea: no significant differences were observed among the groups, indicating consistent ovulation rates before pregnancy. (**E**) Number of implantation sites: the number of implantation sites remained relatively similar across the groups, suggesting that hyperglycemia mainly affected fetal development rather than implantation success. (**F**) Pre-implantation loss (%): increased pre-implantation loss was observed in the STZ-induced hyperglycemic group compared to the control and treated groups. (**G**) Post-implantation loss (%): the STZ-induced hyperglycemic group showed the highest post-implantation loss, while treatment with pandan and teak extracts appeared to mitigate this effect. Groups labeled with different superscript letters denote statistically significant differences (*p* < 0.05; *n* = 5 per group). Data are expressed as the mean ± standard deviation (SD) and were analyzed using one-way ANOVA followed by Tukey’s post hoc test (*p* < 0.05).

As shown in Figure 8A–G, the number of live fetuses was significantly lower in the STZ group compared to the control group (*p* < 0.05). However, a significant increase in live fetuses was observed in the metformin, all the pandan extract doses, and high-dose teak extract groups, with values approaching those of the control group (*p* > 0.05). No significant differences were observed among the groups in the number of dead fetuses, resorptions, corpora lutea, implantation sites, pre-implantation loss, or post-implantation loss (*p* > 0.05).

**Figure 9:**
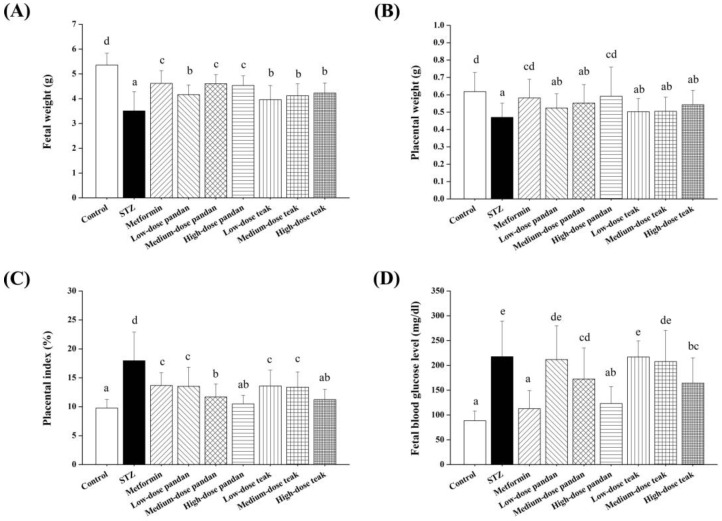
Fetal and placental outcomes in different experimental groups at gestation day 21. (**A**) Fetal weight (g): the streptozotocin (STZ)-induced hyperglycemic group exhibited significantly lower fetal weight compared to the control (*p* < 0.05). Treatment with metformin, pandan, and teak extracts increased the fetal weight, with the medium- and high-dose pandan groups showing the most improvement. (**B**) Placental weight (g): the STZ-induced group had significantly lower placental weight compared to the control (*p* < 0.05). Treatment with metformin and high-dose pandan resulted in improved placental weight, suggesting a protective effect. (**C**) Placental index (%): the placental index (placental weight relative to fetal weight) was significantly higher in the STZ-induced hyperglycemic group than in the control, indicating placental insufficiency. Treatment groups, particularly metformin and medium-dose pandan, showed a trend toward normalization. (**D**) Fetal blood glucose levels (mg/dL): the fetal blood glucose was significantly elevated in the STZ-induced hyperglycemic group compared to the control (*p* < 0.05). Treatment with metformin, medium-dose pandan, and high-dose teak reduced the fetal glucose levels, indicating potential glycemic control benefits. Groups labeled with different superscript letters denote statistically significant differences (*p* < 0.05; *n* = 5 per group). Data are expressed as the mean ± standard deviation (SD) and were analyzed using one-way ANOVA followed by Tukey’s post hoc test (*p* < 0.05).

The newborn weight (Figure 9A) was significantly lower in all the groups compared to the control group (*p* < 0.05). However, the treated groups exhibited significantly higher newborn weights compared to the STZ group (*p* < 0.05), with the highest values recorded in the metformin and medium- to high-dose pandan extract groups.

The placental weight (Figure 9B) was significantly reduced in the STZ group, low-dose and medium-dose pandan extract groups, and all the teak extract groups compared to the control group (*p* < 0.05). Interestingly, the metformin and high-dose pandan extract groups showed significantly higher placental weights, comparable to the control group (*p* > 0.05). The placental index (Figure 9C) was significantly increased in all the groups, except for the high-dose pandan and teak extract groups, compared to the control group (*p* < 0.05).

### 2.7. Blood Glucose Levels in Newborns

The blood glucose levels in newborns (Figure 9D) were significantly higher in all the groups compared to the control group (*p* < 0.05), except for the metformin and high-dose pandan extract groups, which exhibited significantly lower glucose levels compared to the STZ group (*p* < 0.05). However, there were no significant differences among the metformin and extract-treated groups (*p* > 0.05). The blood glucose levels in these groups were significantly lower than those in the STZ group (*p* < 0.05), except in the low-dose pandan and medium- to high-dose teak extract groups, where the glucose levels did not differ significantly from the STZ group (*p* > 0.05).

### 2.8. Dose–Response Analysis of Pandan and Teak Extracts

**Figure 10:**
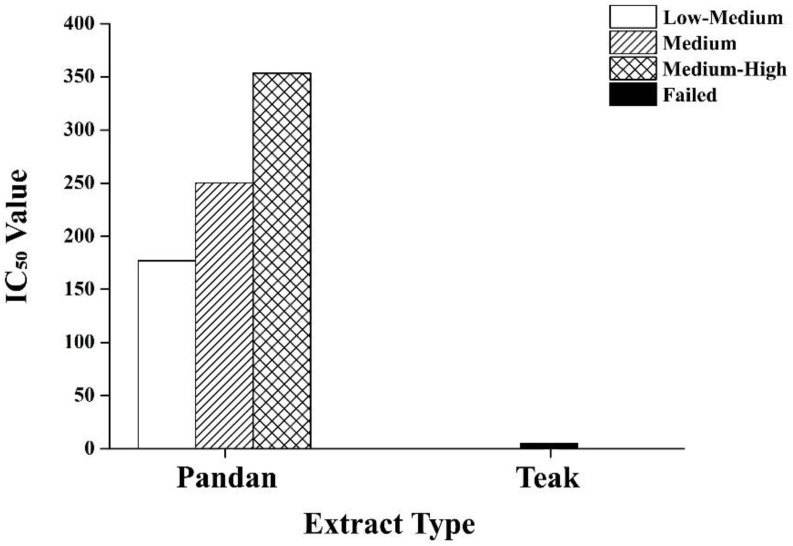
The half-maximal inhibitory concentration (IC_50_) values of the pandan and teak extracts. The pandan extract showed a clear dose-dependent effect, confirming its potential antidiabetic properties. The teak extract failed to produce a measurable IC_50_, suggesting weak or variable bioactivity that may require further optimization or alternative extraction methods.

Figure 10 demonstrates the dose–response analysis of the pandan and teak extracts. A half-maximal inhibitory concentration (IC_50_) for pandan was successfully determined at 176.96–353.52 mg/kg, indicating moderate to high potency. The teak extract did not yield a successful fit curve, suggesting a weaker or inconsistent effect. The pandan demonstrated a dose-dependent reduction in the glucose levels and pancreatic protection, with the high-dose treatment showing effects comparable to metformin. The teak extract had inconsistent responses, with the high-dose treatment showing some benefits, but the lower doses being ineffective.

## 3. Discussion

### 3.1. Efficacy of Metformin and Plant Extracts

Our study confirms that STZ effectively induces hyperglycemia in pregnant rats, validating its use as a model for investigating maternal hyperglycemia during pregnancy. However, we acknowledge that STZ primarily causes beta-cell destruction rather than insulin resistance, which is the hallmark of gestational diabetes mellitus (GDM). Despite this limitation, our model remains valuable for evaluating the hyperglycemia-related pregnancy complications and assessing the potential therapeutic effects of pandan root and teak leaf extracts.

Both metformin and high-dose pandan extract significantly reduced the maternal blood glucose levels by gestation day 21, supporting their antidiabetic potential. This aligns with previous studies demonstrating metformin’s glucose-lowering effects and its relative safety during pregnancy [4]. Similarly, the efficacy of pandan extract in reducing the blood glucose levels correlates with its known bioactive compounds, which have been reported to enhance insulin sensitivity, inhibit α-amylase activity, and improve glucose metabolism [12,21].

While the teak extract also exhibited a glucose-lowering effect, its impact was less pronounced than that of the pandan extract, particularly at lower doses. This difference may stem from variations in the bioactive compound composition or mechanisms of action, which warrant further investigation.

The IC_50_ analysis further confirms the dose-dependent bioactivity of the pandan extract, with higher concentrations demonstrating greater efficacy in glucose regulation and pancreatic protection. In contrast, the failed fit curve for the teak extract suggests that its effects are less predictable, potentially due to weaker bioactivity, limited solubility, or differences in compound stability. The IC_50_ range for pandan is consistent with previous studies highlighting its antidiabetic and antioxidant properties, reinforcing its therapeutic viability as a natural alternative for managing hyperglycemia [22].

### 3.2. Insulin Levels and Pancreatic Morphology

The changes in the insulin levels and pancreatic morphology further support the protective effects of the pandan and teak extracts on beta-cell function. The STZ-treated rats exhibited significantly lower insulin levels and pancreatic mass index, confirming beta-cell damage [23]. Treatment with metformin and high-dose pandan extract resulted in a partial restoration of the insulin levels and pancreatic mass index, suggesting beta-cell preservation or regeneration.

Histopathological analysis (Figure 7) revealed that STZ-induced hyperglycemia led to structural deterioration of the pancreatic islets, contributing to the observed reduction in the islet area. Treatment with metformin and high-dose pandan extract significantly improved the pancreatic morphology, as evidenced by the larger islet areas and better-preserved cellular architecture. These findings align with previous studies demonstrating metformin’s beta-cell protective effects [24].

The dose-dependent effects observed in relation to the pandan extract correlate with improvements in the pancreatic morphology, suggesting a possible beta-cell protective mechanism. Although the teak extract also exhibited some improvement in the pancreatic mass index, its effects were less pronounced than those of the pandan extract. The lack of a consistent dose-response in the teak extract may explain its weaker influence on the insulin levels, potentially due to variability in the bioactive compounds or differences in mechanism of action.

### 3.3. Fetal and Placental Outcomes

Despite the improvements in maternal glucose regulation, the fetal and placental outcomes varied across the treatment groups. The number of live fetuses was significantly lower in the STZ group compared to the controls (*p* < 0.05), indicating that maternal hyperglycemia negatively impacts fetal survival. Treatment with metformin, all the doses of pandan extract, and high-dose teak extract significantly improved the fetal viability, with the values approaching those of the control group.

The reduced newborn weight across all the groups compared to the controls underscores the negative impact of maternal hyperglycemia on fetal growth [25]. This is consistent with previous studies showing that maternal hyperglycemia can lead to fetal growth restriction due to impaired placental function and nutrient transport limitations [26]. Interestingly, the metformin and medium- to high-dose pandan extract groups exhibited the highest newborn weights, indicating a protective effect against fetal growth restriction.

The placental weight was significantly lower in the STZ group and certain extract-treated groups, particularly in the low- and medium-dose pandan extract and all the teak extract groups. This suggests that placental function may have been impaired in these groups. However, the metformin and high-dose pandan extract groups exhibited significantly higher placental weights, comparable to the control values, suggesting improved placental function and nutrient transfer efficiency [27].

### 3.4. Blood Glucose Levels in Newborns

Maternal hyperglycemia has been linked to increased neonatal blood glucose levels, which can predispose offspring to metabolic disorders later in life. Our findings indicate that the blood glucose levels were significantly elevated in newborns from all the groups compared to the controls (*p* < 0.05), except for the metformin and high-dose pandan extract groups, which showed significantly lower neonatal blood glucose levels compared to the STZ group.

These results suggest that high-dose pandan extract may have a protective effect not only on maternal glucose regulation but also on fetal metabolic health, similar to metformin’s well-documented effects in reducing neonatal hyperglycemia [19]. The ability of high-dose pandan extract to mitigate transgenerational hyperglycemia underscores its potential as a natural intervention for reducing metabolic risks in offspring.

### 3.5. Limitations and Future Directions

While our study provides valuable insights into the effects of the pandan and teak extracts on maternal hyperglycemia and pregnancy outcomes, several limitations must be acknowledged to guide future research.

First, we used streptozotocin (STZ) to induce maternal hyperglycemia, which primarily destroys pancreatic beta cells rather than inducing insulin resistance—the hallmark of gestational diabetes mellitus (GDM). Although the STZ model allows for assessment of glucose metabolism and fetal outcomes, it does not fully replicate the pathophysiology of insulin-resistance-driven GDM. Future studies should consider using more relevant models, such as those induced by a high-fat diet (HFD) combined with low-dose STZ or dexamethasone, which better simulate insulin resistance during pregnancy.

Second, our study focused on the short-term maternal and fetal outcomes up to gestation day 21 and did not assess the long-term metabolic effects in offspring. It remains unknown whether maternal treatment with pandan and teak extracts influences postnatal metabolic health, including insulin sensitivity, obesity risk, or diabetes predisposition. Future research should incorporate postnatal follow-up to determine whether these plant-based interventions provide lasting metabolic benefits beyond pregnancy.

Third, although we evaluated the glucose levels, insulin secretion, and pancreatic histology, we did not assess other relevant metabolic biomarkers, such as inflammatory cytokines, oxidative stress markers, or lipid profiles, which are critical for understanding the broader mechanisms underlying glucose regulation. Further investigation into these pathways is warranted to clarify how the pandan and teak extracts exert their antidiabetic effects.

Fourth, while the pandan and teak extracts were tested separately in this study, it remains unclear whether their combination could yield enhanced therapeutic effects through synergism. The inconsistency observed in the dose–response of the teak extract also suggests that future studies should explore optimized extraction methods, bioavailability enhancement, or synergistic combinations to improve the efficacy. Investigating whether the combined administration of pandan and teak extracts produces stronger glucose-lowering effects than either extract alone could help identify effective plant-based treatment strategies.

Fifth, the total phenolic content (TPC) of the pandan and teak extracts was not measured in this study. Given that phenolic compounds are known to contribute significantly to the antioxidant and antidiabetic properties of medicinal plants, quantifying the TPC would help correlate the chemical composition with the observed biological activity. Future studies should include detailed phytochemical analyses, such as phenolic and flavonoid profiling, to strengthen the mechanistic interpretations.

Lastly, although our findings demonstrate promising effects in an animal model, the direct translation of these results to human pregnancy remains uncertain. While some clinical studies have reported glucose-lowering effects of pandan in diabetic patients, further research is needed to validate the efficacy, safety, and appropriate dosing of pandan and teak extracts in pregnant women. Establishing long-term safety profiles and toxicity thresholds is essential before considering these natural agents for clinical use in GDM management.

Despite these limitations, our findings highlight the therapeutic potential of pandan and teak extracts as natural alternatives for managing hyperglycemia during pregnancy. Addressing the outlined research gaps will strengthen the scientific foundation for using these plant-based interventions and enhance our understanding of their role in improving maternal and fetal metabolic health.

## 4. Materials and Methods

### 4.1. Preparation of Extracts

The roots of pandan were collected from the Mueang district in Chachoengsao province, and the leaves of teak were collected from the Mueang district in Rayong province, Thailand. The identification of both plants was confirmed by the Center for Plant Genetic Conservation Project Under the Royal Initiative of Her Royal Highness Princess Maha Chakri Sirindhorn of Suranaree University of Technology (CRSPG SUT) in Nakhon Ratchasima province, Thailand, and voucher specimens were deposited as RSPG. H.B. No. 2304 for pandan and RSPG. H.B. No. 2303 for teak.

The plant extraction processes employed in this study were guided by their reported antidiabetic properties, as supported by previous research. Briefly, the pandan roots and teak leaves were washed, oven-dried at 60 °C for two days, and ground into powder. The dried pandan root powder (2.62 kg) was extracted with 52.5 L of boiled water at 70 °C for 8 h [28]. The dried teak leaf powder (2.75 kg) was extracted four times with 38 L of 70% ethanol [29]. Both extracts were filtered and concentrated by evaporation at 45 °C to a final volume of approximately 400 mL and stored in a cool place throughout the study.

### 4.2. Chemical Analysis of Extracts

Gas chromatography–mass spectrometry (GC/MS) was utilized for phytochemical analysis of the pandan root and teak leaf extracts using an Agilent Technologies 7890A Gas Chromatograph with a 7000B Mass Selective Detector and GC-QQQ software (version B.05.01) (Agilent Technologies, Inc., Santa Clara, California, USA), following the National Institute of Standards and Technology (NIST) mass spectral library search 2.0.

The extracts were dissolved in ethanol and filtered through a 0.45-micron filter in split mode (5:1). Helium gas was used as a carrier at a flow rate of 1 mL/min. The injector temperature was set to 250 °C, and the analytes were separated on a silica capillary column (30 m × 0.20 mm I.D × 0.11 mm film thickness). The initial oven temperature was set at 40 °C and was increased to 280 °C at a rate of 5 °C/min. The mass spectrum determination was performed using an ionization energy of 70 eV. The quantification was based on the mass spectra and retention times [12].

### 4.3. Animal Ethics Statement

All the experimental procedures involving animals were conducted in accordance with the guidelines provided by the Institutional Animal Care and Use Committee (IACUC) of Suranaree University of Technology, Nakhon Ratchasima, Thailand. The protocol (approval number: SUT-IAUCUC-008/2022) was reviewed and approved by the IACUC before the commencement of the study. Special care was taken to minimize discomfort, distress, and pain in the animals throughout the experimental period. The euthanasia procedures were performed according to established guidelines to ensure minimal suffering.

### 4.4. Preparation of Experimental Animals

A total of 135 healthy female Wistar rats (3–6 months old, 250–300 g) were included in this study. Animals exhibiting signs of illness or sterility, or that died during the experimental period, were excluded. The rats were maintained in the Animal Facility of Suranaree University of Technology (SUT) under standardized conditions: ambient temperature of 20–25 °C, relative humidity of 30–70%, and a 12 h light/dark cycle. All the animals had ad libitum access to standard chow and water.

After a one-week acclimatization period, the females were co-housed overnight with males (2:1 ratio). Vaginal smears were collected the next morning between 09:00 and 12:00 a.m. to detect spermatozoa, and the day of positive detection was considered gestation day 0.

Confirmed pregnant rats were randomly allocated into two principal groups: a non-diabetic control group and a diabetic experimental group. The diabetic group was further stratified into seven treatment subgroups, each consisting of 10 to 15 rats [30]. Gestational hyperglycemia was induced on gestation day 5 by a single intraperitoneal injection of streptozotocin (STZ) at a dose of 60 mg/kg body weight, dissolved in 0.1 M cold citrate buffer (pH 6.5) [31].

The blood glucose levels were monitored from tail vein blood samples two days after diabetes induction. Rats with blood glucose levels exceeding 200 mg/dL were classified as hyperglycemic. Only rats with stable hyperglycemia were selected for this study [32].

### 4.5. Animal Experimentation

The concentrations of the pandan and teak extracts administered were 125, 250, and 500 mg/kg, selected based on acute toxicity data reported in previous studies [33,34]. The safe dosage levels for pregnant animals were adjusted in accordance with the Organisation for Economic Co-operation and Development (OECD) guidelines for toxicity testing [35]. Additionally, metformin was administered at a dose of 300 mg/kg, as prior research demonstrated that this dosage effectively improved the pregnancy outcomes in gestational diabetes rat models with insulin resistance [36].

The rats were given the extracts orally once a day via gavage from day 7 to day 21 of gestation [37]. The food intake and body weight changes were recorded daily by calculating the difference between the initial and final food amounts each day. Vaginal bleeding in the dams was monitored daily until delivery.

To safeguard the well-being of the experimental animals, multiple strategies were implemented to minimize pain, distress, and discomfort throughout the study. All the procedures were performed in strict accordance with institutional animal care and use guidelines and were approved by the relevant ethics committee. The animals were handled gently to reduce stress; the intraperitoneal injections were administered using fine-gauge needles to minimize discomfort, and oral gavage was performed carefully to avoid aspiration or injury.

The animals were monitored twice daily for clinical signs of distress, including behavioral changes (e.g., reduced activity, abnormal posture), physical signs (e.g., piloerection, unkempt fur), and physiological indicators (e.g., labored breathing, weight loss). Any observed signs of pain or distress were recorded, and animals exhibiting severe or persistent symptoms received appropriate veterinary care or were humanely euthanized when necessary.

The expected adverse events included mild injection-related discomfort and symptoms associated with the STZ-induced gestational diabetes model. Any unexpected adverse effects were promptly documented and assessed by the research team to determine appropriate interventions.

Predefined humane endpoints were established to prevent unnecessary suffering. These included significant weight loss (>15%), inability to eat or drink, severe respiratory distress, unresponsiveness to external stimuli, or signs of severe pain unrelieved by analgesics. Animals meeting these criteria were humanely euthanized using an overdose of anesthetic agents, followed by a confirmatory secondary method to ensure death.

### 4.6. Blood Glucose Measurement

The fasting blood glucose levels were measured weekly via tail vein puncture using a hemoglucometer (Accu-Chek Performa, Roche Diagnostics Ltd., Bangkok, Thailand). The blood glucose levels (mg/dL) were monitored on gestation days 0, 7, 14, and 21.

### 4.7. Maternal and Fetal Outcome Measurement

After euthanasia on gestation day 21, the gravid uterus containing the fetuses and placentae was excised and weighed. The corpora lutea (CL), implantation sites, embryonic mortality, and live and dead fetuses were carefully counted. The pre-implantation loss rates were calculated as $\frac{Number\;of\;CL-Number\;of\;implantation\;sites}{Number\;of\;CL}\times 100$. The post-implantation loss rates were calculated as $\frac{Number\;of\;implantation\;sites-Number\;of\;live\;fetuses}{Number\;of\;implantation\;sites}\times 100$. The fetuses and placentas were separated and weighed to calculate the placental index as $\frac{Placenta\;weight}{Fetus\;weight}\times 100$ [38]. The blood glucose levels in the newborns were measured using a hemoglucometer, with blood samples collected from the jugular vein.

### 4.8. Maternal Serum Collection and Insulin Level Measurement

Blood samples were collected via cardiac puncture for the insulin level analysis on gestation day 21 after euthanasia. Blood in plain tubes was allowed to clot for 30 min. The tubes were then centrifuged at 2500 rpm for 12 min at −4 °C. Serum was collected and stored in microcentrifuge tubes at −80 °C for further analysis.

The insulin levels were measured using a Rat Insulin ELISA kit (FineTest^®^, Wuhan Fine Biotech Co., Ltd., Wuhan, China), with the optical density (OD) at 450 nm measured by a Tecan Infinite® M Nano Plus microplate reader (Tecan Trading AG, Zurich, Switzerland). The insulin concentrations were calculated using a standard curve based on the reduction in absorbance of the calibrators (excluding calibrator 0) and compared to the concentration using cubic spline regression.

### 4.9. Pancreas Collection and Histopathological Examination

Histopathological examination of the pancreatic tissues was conducted to observe the morphological changes. The pancreas was collected from the rats after euthanasia on gestation day 21 for each experimental group and weighed to determine the pancreatic mass index (weight of pancreas/weight of body) × 100%. The pancreatic tissues were preserved in 10% formalin solution before the histopathological analysis.

Staining was performed using hematoxylin and eosin (H&E) to visualize the tissue structures and cellular morphology. The stained slides were examined using a 3DHISTECH Pannoramic MIDI II slide scanner with Pannoramic Scanner software version 3.0 (3DHISTECH Ltd., Budapest, Hungary). The islet areas were measured using Slide Viewer version 2.7 (3DHISTECH Ltd., Budapest, Hungary).

The pancreatic histological evaluation was performed by an experienced pathologist in a blinded manner. For each animal, the average area of three pancreatic islets was measured from a representative histological section. Quantitative analysis was conducted using one image per rat, with five rats assessed per experimental group [39].

### 4.10. Data Analysis

The data are presented as the mean ± standard deviation (SD), and the fetal classification is expressed as a percentage. In this study, *n* represents the number of animals.

All the data were analyzed using Microcal Origin software version 8.5 (© OriginLab Corporation, Northampton, MA, USA). One-way analysis of variance (ANOVA), followed by Tukey’s post hoc test, was used to compare the mean values among different treatment groups using SPSS version 17.0 (SPSS Inc., Chicago, IL, USA). A probability level of less than 5% (*p* < 0.05) was considered statistically significant.

For the dose–response analysis, the IC_50_ values (half-maximal inhibitory concentration) were determined using nonlinear regression curve fitting in Microcal Origin software (version 8.5). The fit curve success or failure was assessed to evaluate the consistency and potency of the pandan and teak extracts. If the fit curve failed, the IC_50_ value was not reported, indicating an inconsistent or weak response.

## 5. Conclusions

Our study highlights the potential of pandan root and teak leaf extracts, particularly high-dose pandan, in managing maternal hyperglycemia during pregnancy. The dose–response analysis confirms that pandan extract exhibits significant bioactivity in a concentration-dependent manner, with higher doses leading to improved glucose regulation and pancreatic protection. In contrast, teak extract shows an inconsistent dose–response, suggesting weaker or variable effects, which may be influenced by the bioactive compound composition, solubility, or bioavailability.

These natural treatments offer promising alternatives or adjuncts to conventional therapies like metformin, demonstrating notable benefits in blood glucose regulation, pancreatic function preservation, and fetal outcomes. However, the failure of the fit curve for the teak extract indicates that further optimization, alternative extraction techniques, or synergistic combinations may be needed to enhance its efficacy.

While STZ does not fully replicate the insulin resistance characteristic of GDM, our findings provide important insights into the hyperglycemia-related pregnancy complications. Future research should focus on insulin-resistance-based models, exploring the potential synergistic effects between pandan and teak extracts, and assessing the long-term metabolic outcomes to further evaluate their therapeutic potential in pregnancy-associated hyperglycemia.

By advancing our understanding of these dose-dependent effects and refining these natural interventions, we can contribute to the development of safer, more effective plant-based treatments for maternal hyperglycemia and its associated risks.

## Data Availability

The original contributions presented in this study are included in the article, and further inquiries can be directed to the corresponding authors. The data are not publicly available due to the institutional policy on data confidentiality and ethical considerations involving animal research.

## Supplementary Materials

The following supporting information can be downloaded at https://www.mdpi.com/article/10.3390/ijms26125506/s1.

## Abbreviations

The following abbreviations are used in this manuscript:

GDM	Gestational diabetes mellitus
STZ	Streptozotocin
GC/MS	Gas chromatography–mass spectrometry
IR	Insulin resistance
BW	Body weight
SD	Standard deviation
H&E	Hematoxylin and eosin
IC_50_	Half-maximal inhibitory concentration
HFD	High-fat diet
CRSPG SUT	Center for Plant Genetic Conservation Project Under the Royal Initiative of Her Royal Highness Princess Maha Chakri Sirindhorn of Suranaree University of Technology
SUT	Suranaree University of Technology
NIST	National Institute of Standards and Technology
IACUC	Institutional Animal Care and Use Committee
CL	Corpora lutea
OD	Optical density
ANOVA	One-way analysis of variance

## References

[1] Lende M., Rijhsinghani A. (2020). Gestational Diabetes: Overview with Emphasis on Medical Management. Int. J. Environ. Res. Public Health.

[2] Zhang X., Xiao Y. (2019). The Association Between Trimester-Specific Weight Gain and Severe Preeclampsia/Adverse Perinatal Outcome in Gestational Diabetes Mellitus Complicated by Preeclampsia: A Retrospective Case Study. Diabetes Ther..

[3] Mistry S.K., Das Gupta R., Alam S., Kaur K., Shamim A.A., Puthussery S. (2021). Gestational diabetes mellitus (GDM) and adverse pregnancy outcome in South Asia: A systematic review. Endocrinol. Diabetes Metab..

[4] Picón-César M.J., Molina-Vega M., Suárez-Arana M., González-Mesa E., Sola-Moyano A.P., Roldan-López R., Romero-Narbona F., Olveira G., Tinahones F.J., González-Romero S. (2021). Metformin for gestational diabetes study: Metformin vs insulin in gestational diabetes: Glycemic control and obstetrical and perinatal outcomes: Randomized prospective trial. Am. J. Obstet. Gynecol..

[5] Mercado-Méndez S., González-Sepúlveda L., Romaguera J., González-Rodríguez L.A. (2021). The Use of Oral Hypoglycemic Agents during Pregnancy: An Alternative to Insulin?. Puerto Rico Health Sci. J..

[6] Li C., Gao C., Zhang X., Zhang L., Shi H., Jia X. (2022). Comparison of the effectiveness and safety of insulin and oral hypoglycemic drugs in the treatment of gestational diabetes mellitus: A meta-analysis of 26 randomized controlled trials. Gynecol. Endocrinol..

[7] Yedjou C.G., Grigsby J., Mbemi A., Nelson D., Mildort B., Latinwo L., Tchounwou P.B. (2023). The Management of Diabetes Mellitus Using Medicinal Plants and Vitamins. Int. J. Mol. Sci..

[8] Murugan P. (2021). Antidiabetic effect on some medicinal plants. Int. J. Curr. Res. Life Sci..

[9] Chiabchalard A., Nooron N. (2015). Antihyperglycemic effects of *Pandanus amaryllifolius* Roxb. leaf extract. Pharmacogn. Mag..

[10] Sida N.A., Mahmudah R.A., Trinovitasari N., Shofa N., Parawansah, Nuralifah, Rafid A., Risma (2024). *Tectona grandis* Linn.: Antidiabetic activity of the fractions using an in vivo approach. Med. Sains J. Ilm. Kefarmasian.

[11] Vo T.S., Le P.U., Ngo D.H. (2022). In Vitro Hypoglycemic and Radical Scavenging Activities of Certain Medicinal Plants. Exp. Appl. Biomed. Res..

[12] Bhuyan B., Sonowal R. (2021). An overview of *Pandanus amaryllifolius* Roxb. exLindl. and its potential impact on health. Curr. Trends Pharm. Res..

[13] Wang W., Ren Z., Zheng S., Wu H., Li P., Peng W., Su W., Wang Y. (2024). Botany, phytochemistry, pharmacology, and applications of *Pandanus amaryllifolius* Roxb.: A review. Fitoterapia.

[14] Yulianto W., Sulistyani, Swasono F.D.H. (2021). The effect of *Pandanus amaryllifolius* Roxb. extract addition and cooling period on the preference levels, chemical properties and glycemic index of Cr and Mg fortified—Parboiled rice. Food Res..

[15] Poowanna R., Hodruecha N., Kumchoo P., Prathumtet J., Poonsawat J. (2017). Influence of Duration Times of Storage on the Caffeic Acid Contents in *Pandanus amaryllifolius* Root Decoction. Isan J. Pharm. Sci..

[16] Ordas J.A.D., Nonato M.G., Moran C.B. (2020). Ethnobotanical Uses of Pandanaceae Species in Selected Rural Communities in the Philippines. Econ. Bot..

[17] Asdaq S.M.B., Nayeem N., Abida, Alam M.T., Alaqel S.I., Imran M., Hassan E.E., Rabbani S.I. (2022). Tectona grandis L.f: A comprehensive review on its patents, chemical constituents, and biological activities. Saudi J. Biol. Sci..

[18] Balslev H., Phumthum M. (2018). Thai Ethnomedicinal Plants Used for Diabetes Treatment. OBM Integr. Complement. Med..

[19] Zahroh R., Istiroha, Suwanto, Rohmah D.Z. (2023). The influence boiling of fragrant pandan and cinnamon to reducing blood glucose levels in diabetes mellitus. Lux Mensana J. Sci. Health.

[20] Salazar-Petres E.R., Sferruzzi-Perri A.N. (2022). Pregnancy-induced changes in β-cell function: What are the key players?. J. Physiol..

[21] Ningtiyas E.N., Widyaningsih T.D., Sutrisno A. (2019). Optimization of the Formulation of Antidiabetic Functional Drinks According to Sorghum, Red Ginger, and Aromatic Pandan Leaves in Type II Diabetes Rats. Int. Res. J. Adv. Eng. Sci..

[22] Saenthaweesuk S., Naowaboot J., Somparn N. (2016). *Pandanus amaryllifolius* leaf extract increases insulin sensitivity in high-fat diet-induced obese mice. Asian Pac. J. Trop. Biomed..

[23] Gong L., Jiang S., Tian J., Li Y., Yu W., Zhang L., Xiao D. (2024). STZ-induced gestational diabetes exposure alters PTEN/AKT/mTOR-mediated autophagy signaling pathway leading to increase the risk of neonatal hypoxic-ischemic encephalopathy. Reprod. Toxicol..

[24] He X., Gao F., Hou J., Li T., Tan J., Wang C., Liu X., Wang M., Liu H., Chen Y. (2021). Metformin inhibits MAPK signaling and rescues pancreatic aquaporin 7 expression to induce insulin secretion in type 2 diabetes mellitus. J. Biol. Chem..

[25] Paulsen M.E., Brown S.J., Satrom K.M., Scheurer J.M., Ramel S.E., Rao R.B. (2021). Long-term outcomes after early neonatal hyperglycemia in VLBW infants: A systematic review. Neonatology.

[26] Tian M., Reichetzeder C., Li J., Hocher B. (2019). Low birth weight, a risk factor for diseases in later life, is a surrogate of insulin resistance at birth. J. Hypertens..

[27] Salavati N., Smies M., Ganzevoort W., Charles A.K., Erwich J.J., Plösch T., Gordijn S.J. (2019). The Possible Role of Placental Morphometry in the Detection of Fetal Growth Restriction. Front. Physiol..

[28] Reshidan N.H., Abd Muid S., Mamikutty N. (2019). The effects of *Pandanus amaryllifolius* (Roxb.) leaf water extracts on fructose-induced metabolic syndrome rat model. BMC Complement. Altern. Med..

[29] Kusnadi J., Arumingtyas E.L., Ningtyas D.W., Setiawan E.C. (2016). Antioxidant activity of MAE extracted teak (*Tectona grandis* LF) leaves collected from different plantation site at Java Island. Indones. Int. J. ChemTech Res..

[30] Pasek R.C., Gannon M. (2013). Advancements and challenges in generating accurate animal models of gestational diabetes mellitus. Am. J. Physiol. Endocrinol. Metab..

[31] Piazza F.V., Segabinazi E., de Meireles A.L.F., Mega F., Spindler C.D.F., Augustin O.A., Salvalaggio G.D.S., Achaval M., Kruse M.S., Coirini H. (2019). Severe Uncontrolled Maternal Hyperglycemia Induces Microsomia and Neurodevelopment Delay Accompanied by Apoptosis, Cellular Survival, and Neuroinflammatory Deregulation in Rat Offspring Hippocampus. Cell. Mol. Neurobiol..

[32] Bueno A., Sinzato Y.K., Volpato G.T., Gallego F.Q., Perecin F., Rodrigues T., Damasceno D.C. (2020). Severity of prepregnancy diabetes on the fetal malformations and viability associated with early embryos in rats. Biol. Reprod..

[33] Ahmad Shukor N.A.B. (2017). Xanthine Oxidase Inhibitory Activity of *Pandanus amaryllifolius* Roxb. Master’s Thesis.

[34] Hamdin C.D., Prasedya E., Utami S., Galanova D., Cahyo Saputro D., Nurrochmad A., Murwanti R., Jupri A., Sunarpi H. (2019). Acute Toxicity of Indonesian Natural Food Colorant *Tectona grandis* Leaf Extract in Wistar Rats. J. Med. Sci..

[35] OECD Guideline for Testing of Chemicals (1983). Section 4, Test No 415: One-Generation Reproduction Toxicity Study.

[36] Jorquera G., Echiburú B., Crisosto N., Sotomayor-Zárate R., Maliqueo M., Cruz G. (2020). Metformin during Pregnancy: Effects on Offspring Development and Metabolic Function. Front. Pharmacol..

[37] Nguyen-Ngo C., Salomon C., Quak S., Lai A., Willcox J.C., Lappas M. (2020). Nobiletin exerts anti-diabetic and anti-inflammatory effects in an in vitro human model and in vivo murine model of gestational diabetes. Clin. Sci..

[38] Moraes-Souza R.Q., Sinzato Y.K., Antunes B.T., Umeoka E.H.L., Oliveira J.A.C., Garcia-Cairasco N., Karki B., Volpato G.T., Damasceno D.C. (2020). Evaluation of Maternal Reproductive Outcomes and Biochemical Analysis from Wistar Audiogenic Rats (WAR) and Repercussions in Their Offspring. Reprod. Sci..

[39] Matusin A.H.A., Ghani N.I.A., Ahmad N. (2021). Pancreatic islet regenerative capability of *Dillenia excelsa* in alloxan-induced diabetic rats. J. Appl. Pharm. Sci..

