# Supplementary material for: Potential of Pandan Root and Teak Leaf Extracts in Managing Maternal Hyperglycemia During Pregnancy: Comparative Efficacy and Mechanistic Insights
Source: Int J Mol Sci. 2025 Jun 9;26(12):5506. doi: 10.3390/ijms26125506 (PMC12193165; doi:10.3390/ijms26125506)
Supplement: Supplementary file 1 [file ijms-26-05506-s001.zip › ijms-3675495-supplementary.pdf]

Supplementary Material: GC/MS analysis report of pandan and teak extracts with the relevant details.

- Pandan extract

No.	Peak name	Retention Time (min)	Peak area (%)
1	Acetic acid	4.204	6.47
2	2-Propanone, 1-hydroxy-	4.359	0.40
3	2-Propenoic acid	4.745	0.30
4	2-Propenoic acid, 2-hydroxyethyl ester	5.286	0.95
5	2,3-Butanediol	6.224	38.04
6	Furfural	6.690	0.25
7	Propanoic acid, 2,3-dihydroxy-	6.923	1.38
8	2-Furanmethanol	7.174	0.62
9	4-Cyclopentene-1,3-dione	7.659	0.21
10	Butanoic acid, 4-hydroxy-	8.294	0.20
11	1,2-Cyclopentanedione	8.660	0.60
12	2(3H)-Furanone, dihydro-3-methyl-	9.088	0.49
13	5-Methyl furfural	9.362	0.14
14	2(5H)-Furanone, 3-methyl-	9.611	0.69
15	2,4-Dihydroxy-2,5-dimethyl-3(2H)-furan-3-one	9.770	0.30
16	Phenol	9.845	1.17
17	2,5-Dimethyl-4-hydroxy-3(2H)-furanone	11.679	0.19
18	Phenol, 2-methoxy-	11.924	1.26
19	Pyranone	13.234	1.70
20	2,4-Hexadienedioic acid	13.567	0.68
21	Benzoic acid	13.934	0.76
22	5-Hydroxymaltol	14.085	0.18
23	Catechol	14.454	0.61
24	1,2-Ethanediol, 1-(2-furanyl)-	14.983	1.38
25	2-Methoxy-4-vinylphenol	16.186	0.21
26	Phenol, 2,6-dimethoxy-	16.816	0.44

27	1-Dodecanol	18.921	2.59
28	Homovanillic acid	21.443	0.48
29	Dodecyl acrylate	22.140	6.30
30	4-((1E)-3-Hydroxy-1-propenyl)-2-methoxyphenol	22.749	0.69
31	Tetradecanoic acid	23.061	0.37
32	β-Hydroxypropiovanillone	23.740	0.24
33	Acetosyringone	23.779	0.16
34	Pentadecanoic acid	24.358	0.17
35	3,5-Dimethoxy-4-hydroxyphenethylamine	24.663	0.16
36	n-Hexadecanoic acid	25.698	4.62
37	Propanoic acid, 3-mercapto-, dodecyl ester	26.341	1.78
38	Heptadecanoic acid	26.951	0.27
39	Linoleic acid	27.919	1.19
40	Linolenic acid	28.012	1.11
41	Octadecanoic acid	28.374	1.57
42	Eicosanoic acid	31.227	0.14
43	Diisooctyl phthalate	33.571	0.48
44	10,11-Dihydro-10-hydroxy-2,3-dimethoxydibenz(b,f)oxepin	35.120	0.13
45	Heptacosane	36.067	0.37
46	Dehydrodiconiferyl alcohol	42.302	0.22
47	Campesterol	42.695	0.96
48	Stigmasterol	43.142	3.35
49	β-Sitosterol	44.178	3.32
50	Propanoic acid, 3,3'-thiobis-, didodecyl ester	53.013	9.70

- Teak extract

No.	Peak name	Retention Time (min)	Peak area (%)
1	Acetic acid	4.277	3.03
2	2-Propanone, 1-hydroxy-	4.449	0.47
3	2-Propenoic acid	4.878	0.11

4	2-Propenoic acid, 2-hydroxyethyl ester	5.373	0.36
5	Propanoic acid, 2-oxo-, methyl ester	5.969	0.46
6	Glycolaldehyde dimethyl acetal	6.248	1.39
7	Furfural	6.721	0.34
8	Propanoic acid, 2,3-dihydroxy-	6.861	0.79
9	2-Furanmethanol	7.189	0.23
10	2-Propanone, 1-(acetyloxy)-	7.462	0.27
11	4-Cyclopentene-1,3-dione	7.691	0.07
12	Butanoic acid, 4-hydroxy-	8.318	0.14
13	1,2-Cyclopentanedione	8.615	0.22
14	5-Methyl furfural	9.370	0.09
15	2,4-Dihydroxy-2,5-dimethyl-3(2H)-furan-3-one	9.783	0.12
16	Phenol	9.824	0.21
17	Benzyl alcohol	10.928	0.21
18	1-Amino-2,6-dimethylpiperidine	11.114	0.12
19	2(3H)-Furanone, 5-ethoxydihydro-	11.469	0.07
20	2,5-Dimethyl-4-hydroxy-3(2H)-furanone	11.618	0.15
21	Phenol, 2-methoxy-	11.920	0.38
22	Maltol	12.427	0.25
23	Phenylethyl Alcohol	12.491	1.09
24	Pyranone	13.152	0.85
25	Benzoic acid	13.880	0.92
26	Catechol	14.274	1.11
27	Benzofuran, 2,3-dihydro-	14.580	0.56
28	5-Hydroxymethylfurfural	14.787	0.58
29	Indole	15.875	0.56
30	2-Methoxy-4-vinylphenol	16.191	0.76
31	Phenol, 2,6-dimethoxy-	16.810	0.24
32	Vanillin	17.638	0.15
33	Benzene, 1-(bromomethyl)-3-nitro-	18.407	1.66

34	1-Dodecanol	18.926	2.04
35	Dodecyl acrylate	22.142	3.69
36	4-((1E)-3-Hydroxy-1-propenyl)-2-methoxyphenol	22.751	2.23
37	Tryptophol	22.992	0.13
38	Tetradecanoic acid	23.058	0.27
39	Platambin	24.424	0.31
40	Lidocaine	24.700	0.64
41	n-Hexadecanoic acid	25.690	2.72
42	Propanoic acid, 3-mercapto-, dodecyl ester	26.341	1.30
43	Methyl 1,4-dihydroxy-2-naphthoate	27.057	0.14
44	Phytol	27.599	1.74
45	Linoleic acid	27.975	1.01
46	Linolenic acid	28.058	3.83
47	Octadecanoic acid	28.381	1.20
48	Epi-13-Manool	29.306	0.42
49	Catavic acid	30.194	21.22
50	Methyl copalate	31.010	14.14
51	Anticopalic acid	31.539	12.37
52	Enantio-Polyalthic acid	32.580	1.63
53	Glycerol β-palmitate	33.327	0.52
54	Diisooctyl phthalate	33.583	0.60
55	10,11-Dihydro-10-hydroxy-2,3-dimethoxydibenz(b,f)oxepin	35.133	0.19
56	β-Monolinolein	35.786	0.13
57	Butyl 9,12,15-octadecatrienoate	35.892	0.56
58	Pregnenolone	35.991	0.15
59	α-Monostearin	36.244	0.13
60	Squalene	37.533	0.13
61	4,8,13-Duvatriene-1,3-Diol	39.198	0.28
62	Isosesamin	41.769	0.09

63	Dehydrodiconiferyl alcohol	42.300	0.08
64	Stigmasterol	43.118	0.37
65	β-Sitosterol	44.155	0.49
66	Lupeol	45.647	0.25
67	Propanoic acid, 3,3'-thiobis-, didodecyl ester	53.028	7.04
